# The relationship between iron metabolism, stress hormones, and insulin resistance in gestational diabetes mellitus

**DOI:** 10.1038/s41387-020-0122-9

**Published:** 2020-06-08

**Authors:** Yan Feng, Qi Feng, Yuping Lv, Xinna Song, Hongmei Qu, Yangyang Chen

**Affiliations:** 1 Department of Clinical Nutrition, Yuhuangding Hospital Affiliated to Qingdao University, #20 East Yuhuangding Road, Yantai, 264000 China; 2 Department of General Surgery, CPLA No. 71897, #83 Bayi Road, Xi’an, 710000 China; 3 Department of Medical oncology, Yan Tai Zhifu Hospital, Yantai, 260000 China; 4 Department of Obstetrics, Yuhuangding Hospital Affiliated to Qingdao University, #20 East Yuhuangding Road, Yantai, 264000 China

**Keywords:** Nutrition disorders, Endocrine system and metabolic diseases

## Abstract

**Aim:**

To analyze the relationship between iron metabolism index and stress hormones, insulin resistance, and oxidative stress in gestational diabetes mellitus (GDM).

**Methods:**

From January to November 2019, 75 patients with GDM were selected as GDM group, according to age of 1:1; 75 normal pregnant women were selected as Control group. Blood glucose, insulin, stress hormones such as cortisol, norepinephrine (NE), and epinephrine (E), and iron metabolism index such as serum iron, serum ferritin (SF), and transferrin saturation (TS) were measured. Insulin resistance was evaluated by homeostasis model insulin resistance index (HOMA-IR). Multiple linear regression was used to analyze the relationship between iron metabolism index and stress hormones, insulin resistance, and oxidative stress.

**Results:**

The levels of NE, E, serum iron, SF, and TS saturation in the GDM group were higher than Control group (*t* = 3.82, 2.75, 3.14, 6.12, and 3.90, *P* < 0.05, <0.05, <0.05, <0.01, <0.01); HOMA-IR was higher in the GDM group (*t* = 4.92, *P* < 0.01); malondialdehyde (MDA) was higher, while superoxide dismutase (SOD) was lower than Control group (*t* = 5.25, 4.98, both *P* < 0.01). Epinephrine, norepinephrine, cortisol, and serum ferritin were positively correlated (*r* = 0.21, 0.17, and 0.21); epinephrine, cortisol, and transferrin were positively correlated (*r* = 0.12, 0.31). There was a positive correlation between HOMA-IR and SF and TS (*r* = 0.34, 0.34). MDA was positively correlated with SF and TS (*r* = 0.24, 0.29); SOD was negatively related to SF and TS (*r* = −0.12, −0.17).

**Conclusions:**

Iron metabolism index is related to insulin resistance in GDM women. The change in iron metabolism may be involved in the pathogenesis of gestational diabetes caused by stress- adaptive disorder.

## Introduction

Gestational diabetes mellitus (GDM) is defined as any degree of glucose intolerance with onset or first occurrence during pregnancy^1^. According to the International Association of the diabetes and pregnancy study groups (IADPSG), the prevalence of gestational diabetes in China is 9.3%, and even 17.6% in some regions^2^. During pregnancy, affected by placental function, the levels of various steroids, such as prolactin, placental lactogen, estrogen, progesterone, and glucocorticoid, began to rise rapidly at 24–28 weeks of gestation, and reached a peak at 32–34 weeks of gestation, and insulin sensitivity decreased, thus producing significant insulin resistance. In order to maintain the normal blood glucose level, the mother will secrete more insulin. When the secreted insulin cannot fully compensate for the insulin resistance, the blood glucose will rise, resulting in GDM^3^.

Our previous study found that stress-adaptive disorder plays an important role in the pathogenesis of gestational diabetes^4^. The change in stress hormones can also affect the iron transport process; serum iron is positively related to insulin and blood glucose, suggesting that iron overload may be a risk factor for increased risk of diabetes^5,6^. Iron overload may have a close relationship with the risk of hyperglycemia by affecting the degree of oxidative stress injury in the body^7^. The progress of GDM will affect the quality of life of the mother and fetus, but the pathogenesis is not clear^8^. The purpose of this study is to analyze the relationship between iron metabolism index and stress hormones, insulin resistance, and oxidative stress in GDM women, so as to provide new clues for the pathogenesis of GDM.

## Objects and methods

### Objects

From January to November 2019, routine obstetric examination was carried out in Yuhuangding Hospital of Yantai. Pregnant women with 24–28 weeks of gestation were selected as the screening objects. Seventy-five cases of GDM were diagnosed by oral glucose tolerance test (OGTT). At the same time, 1:1 matching was carried out according to the age. The normal blood glucose pregnant women were selected as Control group. According to the standard formulated by the American Diabetes Association (ADA), GDM can be diagnosed as FPG ≥ 5.1 mmol/L, 1-h blood glucose ≥ 10.0 mmol/L, and 2-h blood glucose ≥ 8.5 mmol/L^9^. The blood glucose of pregnant women is higher than any of the following: FPG ≥ 5.1 mmol/L, 1-h blood glucose ≥ 10.0 mmol/L, 2-h blood glucose ≥ 8.5 mmol/L, and GDM can be diagnosed.

GDM women were 25–36 years of age, and they were categorized into four groups: 24–27 years of age (17 GDM women), 27–30 years of age (16 GDM women), 30–33 years of age (20 GDM women), and 33–35 years of age (22 GDM women), and pregnant women were invited to participate in the control group if they match the age of GDM group. Inclusion and exclusion criteria: pregnant women are the first singleton pregnancy, excluding organ diseases such as heart, liver, spleen, lung and kidney, thyroid disease, pancreatitis, cholecystitis, etc., without family history of diabetes, prepregnancy diabetes, hypertension, polycystic ovarian syndrome and other diseases, and FPG in early pregnancy is <5.6 mmol/L, no hormone or other effects on glycosylation in pregnancy. Other diseases, such as anemia and infection, may affect serum iron and ferritin that are excluded. The study protocol was approved by Yantai Yuhuangding Hospital Ethics Committee, approval number is YYY201916, and all pregnant women included in this study were informed of the purpose of the study and signed informed consent.

## Research methods

### Maternal characteristics

Record maternal age, gestational age at screening, height and weight before pregnancy of two groups, and calculate the body mass index (BMI) before pregnancy = weight (kg)/height (m^2^).

### Stress hormones, iron metabolism indexes, and oxidative stress

After 8–10 h of fasting overnight, the pregnant women who had good sleep in the previous night sat in the rest room of the hospital at 8:00 in the morning for 30 min, and all the venous blood was collected at 8:30 in the morning, and then 75 g of OGTT was performed. The fasting venous blood was reserved for the detection of fasting blood glucose, insulin, stress hormones, and iron metabolism index. Glucose oxidase method was used to measure fasting, 1 and 2 h of blood glucose; electrochemiluminescence method was used to measure serum insulin and cortisol levels; radioimmunoassay was used to measure epinephrine (E) and noradrenaline (NE) levels in patients, all of which were Roche’s matching reagents; homeostasis model insulin resistance index (HOMA-IR) was used to evaluate insulin resistance, HOMA-IR = Fins (mU/L) × FPG (mmol/L)/22.5; in order to eliminate anemia, it is necessary to detect the hemoglobin (Hb) in patients. Hb was detected by Sysmex automatic blood cell analyzer, serum iron (Fe) by ELISA, total iron-binding capacity (TIBC), ferritin (SF) by immunoturbidimetry, and transferrin saturation (TS) = Fe/TIBC × 100%. Malondialdehyde (MDA), superoxide dismutase (SOD), and glutathione (GSH) were used as markers of oxidative stress injury. All markers were detected according to the instruction of the kits (Jiancheng Bioengineering Institute, Nanjing, China). The results are expressed as μmol/L, U/mL, and mg/L.

### Statistical analysis

SPSS 19.0 statistical analysis software was used to analyze our data, which were represented by mean ± standard error of the mean. The comparison of the mean between two groups was conducted by two independent samples’ T test, and the data of non-normal distribution were compared after logarithmic transformation. The correlations of SF, TS with stress hormones, insulin resistance, and oxidative stress were analyzed by multiple linear regression. All hypothesis tests were performed bilaterally. *P* < 0.05 was statistically significant.

## Results

### Maternal characteristics in GDM women

Compared with Control group, the BMI of GDM group before pregnancy was 24.8 ± 2.9 kg/m^2^, which was higher than that of Control group, and the difference was statistically significant (*P* < 0.05). There was no significant difference in maternal age and gestational age at screening (*P* > 0.05) (Table 1).

**Table 1:** Maternal characteristics in GDM women.

Maternal characteristics	Control	GDM
Maternal age (years)	29.4 ± 4.3	28.8 ± 5.1
Gestational age at screening (weeks)	25.3 ± 2.7	26.5 ± 2.0
Pregestational BMI (kg/m^2^)	23.4 ± 3.0	24.8 ± 2.9^a^

Values are expressed as mean ± standard error.

*BMI* body mass index.

^a^Indicates *P* < 0.05 vs. Control group.

### Comparison of stress hormones and oxidative stress in GDM women

#### Stress hormones

Compared with Control group, the cortisol content in GDM group is slightly higher, but the difference is not statistically significant (*P* > 0.05); E and NE in GDM group are increased, and the difference is statistically significant (both *P* < 0.05) (Table 2).

**Table 2:** Comparison of stress hormones and oxidative stress in GDM women.

Groups	Stress hormones			Oxidative stress		
	E (ng/L)	NE (ng/L)	Cortisol (nmol/L)	MDA (μmol/L)	SOD (U/mL)	GSH (mg/L)
GDM	345.8 ± 78.4^a^	178.4 ± 46.6^a^	448.2 ± 100.2	10.3 ± 3.1^b^	87.3 ± 29.8^a^	28.1 ± 8.9
Control	211.3 ± 59.4	117.3 ± 52.8	395.8 ± 137.1	8.4 ± 2.8	123.0 ± 42.1	39.1 ± 12.6
*t*	3.81	2.75	1.42	5.25	4.98	1.06
*P*	0.00	0.03	0.09	0.00	0.00	0.22

*E* epinephrine, *NE* noradrenaline, *MDA* malondialdehyde, *SOD* superoxide dismutase, *GSH* glutathione.

Values are expressed as mean ± standard error.

^a^Indicates *P* < 0.05 vs. Control group.

^b^Indicates *P* < 0.01 vs. Control group.

#### Oxidative stress

Compared with Control group, MDA in GDM group is increased (*P* < 0.01); SOD is decreased in GDM group, and the difference is statistically significant (*P* < 0.01), and GSH is decreased slightly, but the difference is not statistically significant (*P* > 0.05) (Table 2).

#### Comparison of Hb, iron metabolism index, and HOMA-IR in GDM women

Compared with Control group, Hb did not differ between two groups (*P* > 0.05); HOMA-IR is higher in GDM group (*P* < 0.01); Fe, SF, and TS are higher in GDM group than in Control group (*P* < 0.05) (Table 3).

**Table 3:** Comparison of hemoglobin, iron-related indexes, and HOMA-IR in GDM women.

Groups	Fe (μmol/L)	SF (μg/L)	TS	Hb (g/L)	HOMA-IR
GDM	12.3 ± 2.4^a^	184.6 ± 42.9^b^	0.41 ± 0.1^b^	126.4 ± 4.8	2.6 ± 0.5^b^
Control	9.1 ± 3.1	131.1 ± 22.8	0.33 ± 0.2	119.1 ± 8.5	2.1 ± 0.2
*t*	3.14	6.12	3.90	0.88	4.92
*P*	0.05	0.00	0.00	0.45	0.00

*SF* serum ferritin, *TS* transferrin saturation, *Hb* hemoglobin, *HOMA-IR* homeostasis model assessment for insulin resistance.

Values are expressed as mean ± standard error.

^a^Indicates *P* < 0.05 vs. Control group.

^b^Indicates *P* < 0.01 vs. Control group.

### Relationships between iron metabolism index and stress hormones, oxidative stress, and HOMA-IR in GDM women

#### Relationships of iron metabolism index with stress hormones

Multiple linear regression was used to analyze the relationship between iron index and stress hormones. E, NE, and cortisol are dependent variables; after BMI correction, the results showed that SF is positively related to E, NE, and cortisol (*P* < 0.05) in GDM group; the same trend occurred in Control group, but there is no significant difference (*P* > 0.05) (Table 4).

**Table 4:** Relationships between iron metabolism index and stress hormones, oxidative stress, and HOMA-IR in GDM women.

	Groups	Stress hormones						HOMA-IR		Oxidative stress					
		E		NE		Cortisol				MDA		SOD		GSH	
		*r*	*P*	*r*	*P*	*r*	*P*	*r*	*P*	*r*	*P*	*r*	*P*	*r*	*P*
SF	GDM	0.21	0.01	0.17	0.03	0.21	0.00	0.34	0.01	0.24	0.00	−0.15	0.02	−0.12	0.03
	Control	0.14	0.07	0.13	0.21	0.14	0.06	0.22	0.04	0.16	0.02	−0.21	0.19	−0.21	0.06
TS	GDM	0.23	0.02	0.16	0.17	0.31	0.03	0.34	0.00	0.29	0.01	−0.11	0.01	−0.17	0.12
	Control	0.21	0.03	0.16	0.21	0.32	0.04	0.30	0.04	0.32	0.21	−0.17	0.12	−0.12	0.10

*E* epinephrine, *NE* noradrenaline, *MDA* malondialdehyde, *SOD* superoxide dismutase, *GSH* glutathione, *SF* serum ferritin, *TS* transferrin saturation, *HOMA-IR* homeostasis model assessment for insulin resistance.

TS is positively related to E and cortisol in two groups (*P* < 0.05); the same trend occurred in TS and NE in both groups, but there is no significant difference (*P* > 0.05) (Table 4).

#### Iron metabolism index and HOMA-IR

Multiple linear regression was used to analyze the relationship between iron index and HOMA-IR; after BMI correction, the results showed that SF is positively related to HOMA-IR in two groups (*P* < 0.05); the same trend occurred in TS and HOMA-IR (*P* < 0.05) (Table 4).

#### Iron metabolism index and oxidative stress

Multiple linear regression was used to analyze the relationship between iron index and oxidative stress; after BMI correction, the results showed that SF is positively related to MDA, while SOD and GSH are negatively related to SF in GDM group (*P* < 0.05); the same trend occurred in Control group, but only the relationship of MDA and SF is significantly different (*P* < 0.05) (Table 4).

TS is positively related to MDA, and negatively related to SOD in GDM group only (*P* < 0.05) (Table 4).

## Discussion

With the increase in gestational age, the levels of estrogen, progesterone, cortisol, and other hormones in pregnant women gradually increased, insulin resistance increased, hyperglycemia threshold decreased, and the incidence of gestational diabetes gradually increased^10,11^. The results of this study showed that the levels of stress hormones E and NE were all increased, and positively correlated with the level of SF and TS; the content of free cortisol in GDM patients did not change, which was consistent with Kirwan’s study, which may be due to the increase in synthetic corticosteroid-binding globulin in the liver of pregnant women, resulting in the increase of binding cortisol and the decrease in free cortisol, while the decrease in cortisol stimulated adrenocortical stimulation by negative feedback^12^. The final result of hormone secretion will lead to the continuous increase in bound cortisol, while the content of free cortisol has no significant change. The results are consistent with those of previous studies^13,14^. These stress hormones (E and NE) increased in GDM women, and indicated that stress-adaptation disorder may be involved in the occurrence of hyperglycemia; this conclusion is consistent with our previous research results^15^.

On the other hand, we pay more and more attention to the relationship between iron overload and the occurrence and development of diabetes. Most studies believe that iron overload can lead to oxidative stress damage in the body, thus increasing the risk of diabetes^16,17^. On the other hand, iron is mainly deposited in the liver, and liver is an important organ of glucose metabolism; hepatic iron loading will increase the burden of liver and makes the liver work harder, thus inducing glucose dysregulation^18^. The development of diabetes will also promote the deposition of iron, resulting in the increase of ferritin. Ferritin is an indicator of iron reserve; the increase of ferritin is related to the abnormal glucose tolerance, which means that excessive iron storage can promote the pathogenesis of GDM^19,20^. The results of this study showed that serum iron, serum ferritin, and transferrin saturation in GDM patients increased, which may be due to the continuous hyperglycemia of GDM patients affecting the change in iron metabolism, and promoting the deposition of iron in the body.

Ferritin is a storage form of iron and an indicator of inflammation. Increased ferritin level during pregnancy was related to the lower stress state in pregnancy^21,22^. In our study, there was a positive correlation between the stress-adaptation disorder and serum ferritin level in GDM women, which was consistent with the research.

As iron overload also has a direct impact on adrenal function, it may cause changes in some stress hormones. Previous studies found that the increase in iron storage during pregnancy in GDM women may cause the change in adrenal function, leading to the increase in stress hormones’ secretion, thus aggravating the original stress-adaptive disorder and further increasing blood glucose^4^. Elevated ferritin is often used as a marker of iron overload, and oxidative stress induced by iron overload can directly damage islet β cells, thus affecting islet function and leading to hyperglycemia^23,24^. GDM women have a higher level of oxidative stress injury, and the increase in iron storage may be one of the important reasons for the increase in oxidative stress injury, and ferritin was used as a marker to assess iron stores and it is also an acute-phase reactant; our study also found that ferritin was increased slightly and related to insulin resistance.

The lipid peroxidation caused by the increase in iron storage will reduce the utilization of sugar in muscle tissue, increase gluconeogenesis, and cause insulin resistance. This study also found that SF and TS were positively correlated with MDA, negatively related to SOD, and positively correlated with HOMA-IR, which suggested that the increase in iron storage may aggravate oxidative stress injury and promote the occurrence of stress-adaptive disorder, thus increasing insulin resistance and promoting the occurrence and development of GDM. Some studies have found that iron deficiency can increase glycosylated hemoglobin and blood glucose^25^. In addition, other studies have shown that iron deficiency can reduce the prevalence of GDM^26^. However, the lack of large-scale randomized controlled trials has not yet reached a conclusion. Our results only suggest that oxidative stress injury and stress-adaptive disorder related to iron overload may play an important role in the occurrence of GDM.

The occurrence of gestational diabetes mellitus is a very complex process, which is affected by many factors in its development. However, the specific factors involved in the pathophysiological process of GDM and the principle of molecular mechanism are not clear at present. Stress- adaptive disorder may be one of the important factors leading to the occurrence and development of gestational diabetes mellitus, and the change in iron metabolism may play an important role in its occurrence and development. In a large-scale trial in Finland on the effect of iron supplementation on GDM, the results showed that 100 mg daily of iron supplementation for iron-replete women did not increase the risk of hyperglycemia^27^. More clinical trials and in vitro studies are needed to analyze the role of iron in insulin signaling.
